# Evaluation of the Contribution of Multiple DAMPs and DAMP Receptors in Cell Death-Induced Sterile Inflammatory Responses

**DOI:** 10.1371/journal.pone.0104741

**Published:** 2014-08-15

**Authors:** Hiroshi Kataoka, Hajime Kono, Zubin Patel, Kenneth L. Rock

**Affiliations:** 1 Department of Pathology, UMass Medical School, Worcester, Massachusetts, United States of America; 2 Department of Internal Medicine, Teikyo University School of Medicine, Tokyo, Japan; University Paris Sud, France

## Abstract

When cells die by necrosis *in vivo* they stimulate an inflammatory response. It is thought that this response is triggered when the injured cells expose proinflammatory molecules, collectively referred to as damage associated molecular patterns (DAMPs), which are recognized by cells or soluble molecules of the innate or adaptive immune system. Several putative DAMPs and/or their receptors have been identified, but whether and how much they participate in responses *in vivo* is incompletely understood, and they have not previously been compared side-by-side in the same models. This study focuses on evaluating the contribution of multiple mechanisms that have been proposed to or potentially could participate in cell death-induced inflammation: The third component of complement (C3), ATP (and its receptor P2X7), antibodies, the C-type lectin receptor Mincle (Clec4e), and protease-activated receptor 2 (PAR2). We investigate the role of these factors in cell death-induced inflammation to dead cells in the peritoneum and acetaminophen-induced liver damage. We find that mice deficient in antibody, C3 or PAR2 have impaired inflammatory responses to dying cells. In contrast there was no reduction in inflammation to cell death in the peritoneum or liver of mice that genetically lack Mincle, the P2X7 receptor or that were treated with apyrase to deplete ATP. These results indicate that antibody, complement and PAR2 contribute to cell death-induced inflammation but that Mincle and ATP- P2X7 receptor are not required for this response in at least 2 different *in vivo* models.

## Introduction

Over the last decade it has become apparent that the immune system not only provides continual surveillance for microbes, but also monitors the health of the host's own cells [1], [2]. This is well illustrated by the immune response to cell injury. When cells die by necrosis, this event is sensed by the innate and adaptive immune system and responses are initiated [3]–[5]. This occurs regardless of the underlying cause of cell death, including when it is due to a sterile process, such as an ischemic or toxic insult [6].

One of the early and important responses to cell death is acute inflammation [7]. Upon recognizing cell death, tissue resident macrophages are stimulated to produce the cytokine IL-1, which is a key mediator in recruiting leukocytes to the site of injury [8], [9]. Within hours after cell injury, neutrophils begin to extravasate into the tissue and this process accelerates over a period of 24 hours. With somewhat delayed kinetics monocytes also emigrate into the tissue where they differentiate into macrophages [10], [11].

The inflammatory response to cell death subserves a number of biological functions and can have both positive and negative consequences [3]–[5]. On the one hand it rapidly delivers cellular and soluble defenses to the site of death and this may neutralize or contain the injurious process [12]. It also helps to clear debris and stimulate repair. On the other hand, the recruited leukocytes release anti-microbial molecules, such as reactive oxygen species and proteases, that can damage the tissues [12]. Moreover, the inflammatory mediators that are produced can stimulate processes such as fibrosis that can interfere with tissue regeneration and function. As a result of these many effects, the inflammatory response to cell death can contribute to host defense and/or cause disease. Consequently, cell death-induced inflammation is medically important.

Exactly how cell death stimulates inflammation is incompletely understood. When cells die, there is clearly a change that occurs which converts them from a non-phlogistic state to one that is proinflammatory. It is thought that a key event in this metamorphosis is the loss of integrity of the plasma membrane [4]. Rupture of the plasmalemma is a hallmark of necrosis and when it occurs intracellular molecules are exposed [13]. It is thought that the innate immune system has evolved mechanisms to recognize some of these normally hidden molecules (damage associated molecular patterns (DAMPs)) directly or some of the products generated by these molecules, e.g. hydrolysis products from released cellular proteases [3], [4].

The molecular identity of DAMPs and the contribution to inflammation of various of these molecules is incompletely understood [14]. Among the putative DAMPs thus far identified are both cellular proteins, such as HMGB1, IL-1 and SAP130, and nucleic acid-related molecules, such as DNA, ATP and uric acid [15]–[21]. It has also been suggested that cell death could trigger inflammation by activating the complement cascade directly or secondarily after natural antibodies bind to cellular constituents [22].

There are also a number of cells and cellular receptors that could be involved in sensing cell death and triggering inflammation. The C-type lectin receptor Mincle (Clec4e), which is expressed on cells of the innate immune system, has been implicated stimulating cell death-induced inflammation [21]. In addition, proteases released from dying cells could potentially cleave the protease-activated receptor 2 (PAR2), which when hydrolyzed stimulates cytokine production and inflammation, although a role for PAR2 in cell death-induced inflammation has not been previously examined [23].

However, the importance of these various mechanisms to cell death-induced inflammation is not clear. Depletion of uric acid and ATP and antibody blockade of Mincle have been reported to reduce inflammatory responses to cell death *in vivo*, although their contribution to this process has not been compared in the same models [18], [19], [21]. In contrast, loss of some of these other putative DAMPS (HMGB1) in dead cells does not reduce the inflammatory response at least in some experimental systems and the role of other triggering mechanisms has not been evaluated *in vivo* [8]. The present studies were initiated to evaluate the role of many of these triggers and to compare them in the same experimental systems. We show that complement, antibody and PAR2 contribute to cell death-induced inflammatory responses.

## Materials and Methods

### Reagent and antibodies

Antibodies against Ly-6G (clone 1A8) were obtained from BD Bioscience. Anti- 7/4 antibody was purchased from Serotec. 7-AAD was obtained form Molecular probe. Acetaminophen, ATP (A7699) and Apyrase (grade VII) were purchased form Sigma. ATP quantification kit (CA10) was purchased from Wako Pure Chemical Industries, Ltd.

### Animal and cell lines

Wild type C57BL/6, P2X7 receptor-deficient mice [24], C3-deficient mice [25], PAR2-deficient mice (*F2rl1^tm1Mslb^*) [26] were purchased from Jackson Laboratories or Japan SLC, Inc. Mincle deficient mice [27] were obtained form Consortium for Functional Glycomics at The Scripps Research Institute. µMT (B cell-deficient) mice (*Igh-6^tm1Cgn^*) were provided by Dr. Susan Swain (UMass Medical School). All animal protocols were approved by the UMass and Teikyo University animal care and use committee. EL4 cells were maintained in RPMI-1640 with 10% fetal calf serum (FCS) and antibiotics. Mice were humanely euthanized by either of CO_2_ narcosis or inhalation of overdose isofluorane before harvesting tissues in all experiments.

### Preparation of necrotic cells and apyrase treatment

Necrotic EL4 cells were prepared as described [9]. EL4 cells were washed 5 times with phosphate buffered saline (PBS), then resuspended in PBS at 10 million cells /50 µL and subsequently heat-shock at 45°C for 10 min followed by 37°C incubation for 5 hours; this resulted in necrosis (7-AAD/PI positive cells).

To induce mechanical necrosis, liver from C57BL/6 was weighed, mixed with 5 times of weight of PBS and subjected to mechanical injury by a dounce homogenizer followed by nitrogen cavitation for 10 min at 500 psi. Similarly, mechanical necrosis was induced in EL4 by ultrasonic sonication for 30 seconds. In some experiments, mice were injected with 25 units of intact or boiled (95°C for 5 min) apyrase s.c. and 30 minutes later challenged with acetaminophen, as described below, or apyrase i.p. and immediately followed by the i.p. injection with dead cells.

### Serum transfer and quantitative detection of serum total IgG

Blood was collected from normal C57BL/6 mice and allowed to clot in a 1.7 ml tube and then serum was separated by centrifugation at 8000 rpm for 10 minutes. Serum was stored at −80°C after filtration in 0.22 µm Spin-X tube (Costar). Control or µMT mice were injected i.v. with 500 µL serum one day before dead cells were injected or acetaminophen treatment. To confirm the efficiency of serum transfer, at the time of peritoneal lavage or liver harvest serum was taken from each mouse and total IgG was measured by Mouse IgG Total ELISA kit according to the manufacturer's protocol (eBioscience).

### Neutrophil recruitment to peritoneal cavity

Quantification of recruited neutrophils and monocytes to the peritoneal cavity was described before [9]. Mice were injected i.p. 10 million or 30 million of necrotic EL4 cells in 150 µL of PBS, or 150 µL of liver homogenate. After 4 or 16 hours of injection, the peritoneum was lavaged with 6 mL of PBS with 2%FCS, 3 mM of EDTA and 10 U/mL of heparin. The absolute number of neutrophil (Ly-6G^+^ 7/4^+^) in 100 µL of lavage was determined using a flow cytometer equipped with a high throughput volumetric sampler (BD bioscience and Sony Biotechnology).

### Acetaminophen induced liver injury

Acetaminophen (Sigma) was dissolved at the concentration of 15 mg/ml in PBS heated at 55°C. Mice were fasted for 18 hours in advance of acetaminophen treatment and injected i.p. with 300 mg/kg of acetaminophen. The treated mice were provided food 4 hours later. 18 hours after acetaminophen injection the mice were humanely sacrificed and their livers were perfused with HBSS introduced through the inferior vena cava and harvested. Neutrophilic responses to acetaminophen in liver were initially assessed by two methods, myeloperoxidase assay and quantifying neutrophils by flow cytometry. These two assays were found to give concordant results and therefore in some of the models, responses were assayed only by flow cytometry. Myeloperoxidase assay was done as follows; one hundred mg of liver was homogenized in 1 mL of myeloperoxidase buffer (0.5% hexadecyl trimethyl ammonium bromide, 10 mM EDTA, 50 mM Na_2_HPO_4_, pH 5.4), sonicated and assayed for myeloperoxidase activity as described [8] To count immune cell infiltration, the harvested livers were treated with a buffer containing 0.05% collagenase IV (Sigma), 0.028% DNase I (Sigma), 1.25 mM CaCl_2_ and 4 mM MgCl_2_ in HBSS buffer (Gibco) at 37°C. Nonparenchymal cells were then isolated from whole liver cells in 50% OptiPrep density gradient medium (Sigma) diluted with RPMI media and stained with 7/4 FITC, Ly6G PE, CD11b Cy5.5 and F4/80 APC antibodies at 1∶100 concentration each in 2.4G2 supernatant. Recruited inflammatory cells were counted on BD High Throughput Sampler-installed FACSCalibur (BD).

### Statistical analyses

Data are reported as means ± standard errors. The distribution of data was judged by D'Agostino and Pearson omnibus normality test. Statistical analyses in each independent experiment was performed with an unpaired, two-tailed Student's *t*-test. One - way ANOVA and Dunnett's multiple comparison post-test were used to compare the means of multiple groups to the control group. *P*<0.05 was considered statistically significant.

## Results

### Role of Mincle (Clec4e) in cell death-induced inflammation

An earlier study implicated the C-type lectin receptor Mincle in the death-induced inflammatory response [21]. In this study, cell death was induced in the thymus by irradiating mice and this stimulated a neutrophilic inflammatory response. This response was attenuated by the systemic administration of an anti-Mincle mAb. This finding suggested that Mincle was an important receptor for death-induced inflammatory response. However, Mincle-null mice have not been examined.

**Figure 1:**
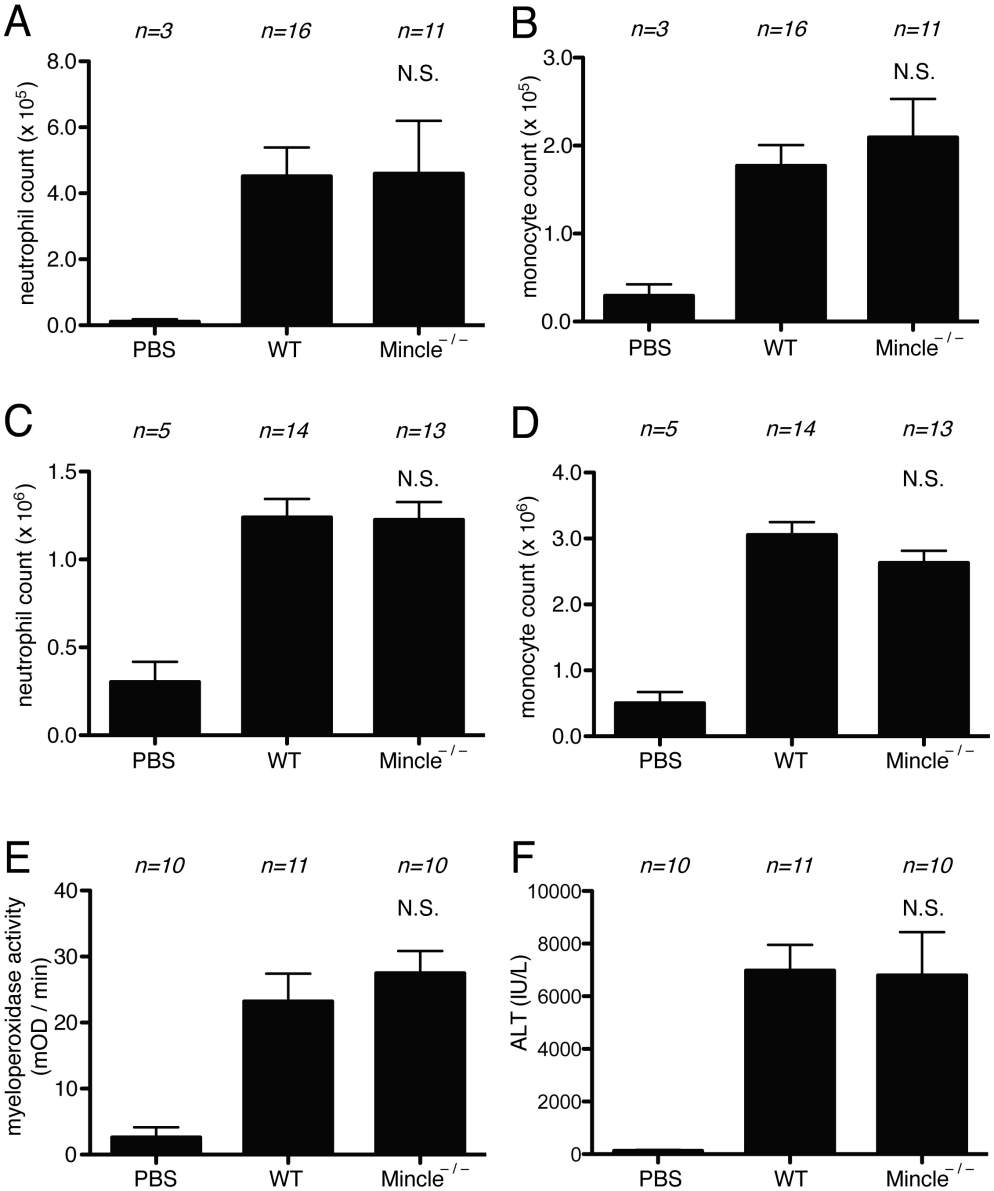
Role of Mincle in cell death induced inflammatory responses. (A, B, C, D) The total neutrophil (A, C) and monocyte (B, D) numbers in the peritoneal cavity of WT C57BL/6 mice and Mincle-deficient mice 4 hours (A, B) or 16 hours (C, D) after i.p. injection of heat-shocked necrotic EL4 cells. (E, F) Myeloperoxidase activity in the liver (E) and serum levels of ALT (F) of mice challenged with i.p. injection of 300 mg/kg of Acetaminophen in C57BL/6 or Mincle- deficient mice. The data are combined results of 3 experiments and represented as means ±SEM (*n* =  total number of mice from the multiple experiments for each group). NS, not significant versus WT groups. PBS, WT mice injected with PBS.

To assess the role of Mincle in death-induced inflammatory responses, we analyzed mice that were genetically deficient in Mincle [27]. The Mincle-deficient mice were injected i.p. with necrotic EL4 cells and their resulting neutrophilic inflammatory response was quantified and compared to those in wild type animals. Neutrophils could be detected extravasating into the peritoneum of wild type mice within 4 hours of the dead cell injection (Fig 1A). In these experiments, there was no reduction in neutrophilic inflammatory responses in the mutant mice as compared to wild type ones after 4 hours. We also investigated neutrophil accumulation at the peak (16 hr) of responses and these responses were also equivalent between the wild type and Mincle-mutant mice (Figure 1A, B, C, D).

To extend these results to another model, we treated Mincle-deficient and wild type mice with a dose of acetaminophen that causes liver cell necrosis and then 16 hour later quantified neutrophil in their livers. Few neutrophils are present in the livers of untreated mice, but their numbers significantly increased in the livers injured by acetaminophen, as expected (Figure 1E, F). In these assays the Mincle-deficient mice developed as much neutrophilic inflammation as the wild type ones. Therefore, we find that Mincle is not required for cell death-induced inflammatory responses in two distinct assays systems.

### Role of PAR2 in cell death-induced inflammation

Protease-activated receptor 2 (PAR2) is a G protein-coupled receptor that is expressed on the surface of most leukocytes as well as other cell types [28]. This receptor is stimulated when its ectodomain is cleaved by a variety of proteases including ones from the host, such as trypsin, tryptase and protease 3, and others from exogenous sources such as bacteria or allergens [28]. In this process, the proteolytic cleavage of the receptor liberates a fragment that binds to and stimulates the cleaved PAR2 receptor. When PAR2 is thus stimulated, it activates the expression of a number of genes that are involved in inflammation, including cytokines and adhesion molecules. When PAR2 peptide agonists or proteases that activate this receptor are injected *in vivo*, they stimulate inflammation [29]–[31]. The role of PAR2 in the cell death-induced sterile inflammatory response has not been previously investigated.

**Figure 2:**
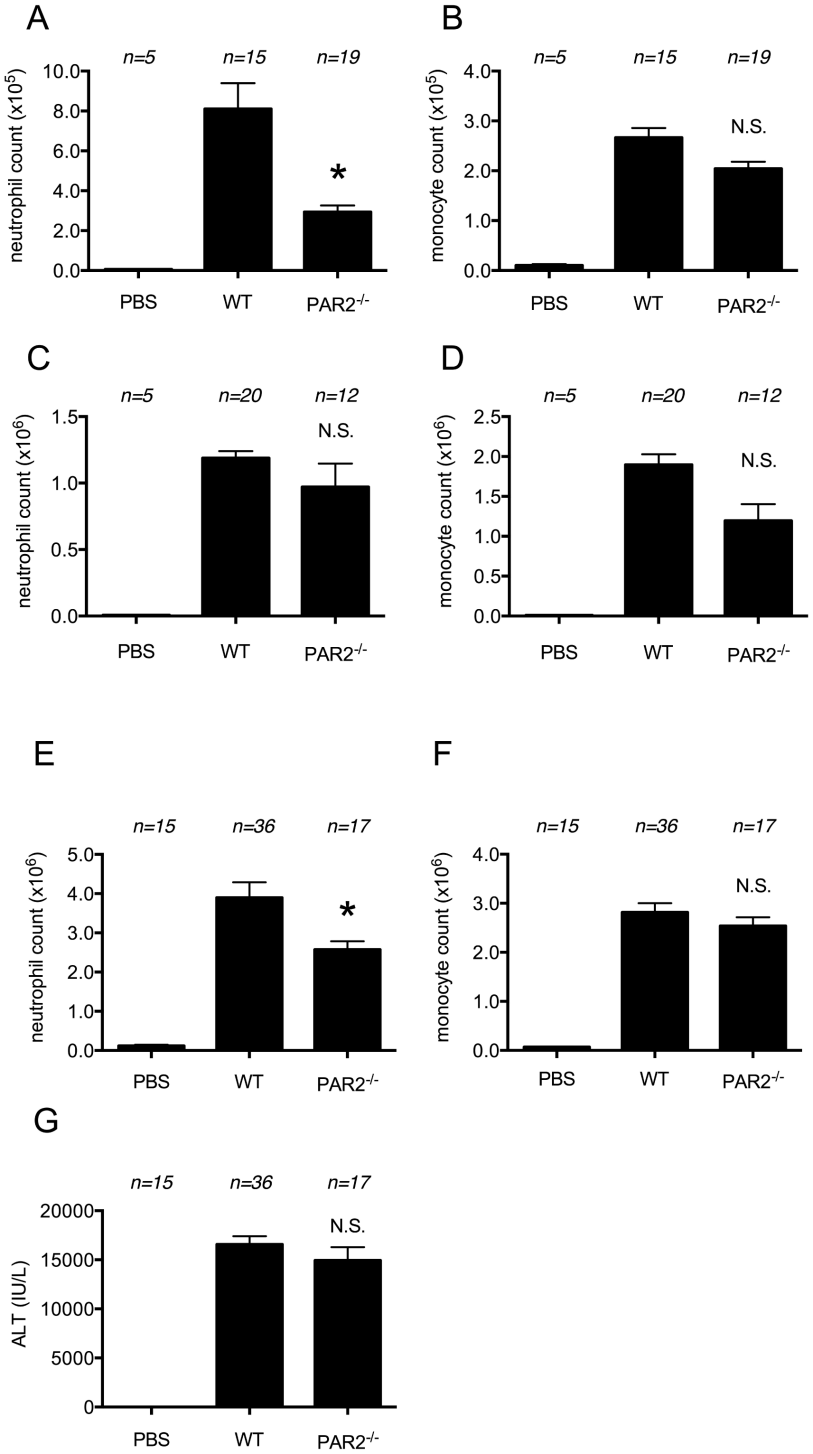
Role of PAR2 in cell-death induced inflammation. (A, B) The total neutrophil (A) and monocyte (B) numbers in peritoneal cavity of WT C57BL/6 mice and protease-activated receptor 2 (PAR2)-deficient mice 4 hours after i.p. injection of heat-shocked necrotic EL4 cells. (C, D) The total neutrophil (C) and monocyte (D) numbers in peritoneal cavity of WT C57BL/6 mice and µMT mice 16 hours after i.p. injection of heat-shocked necrotic EL4 cells. The data are combined results of 5 experiments and represented as means ±SEM (*n* =  total number of mice from the multiple experiments for each group). (E, F, G) The total neutrophil (E) and monocyte (F) numbers in hepatic nonparenchymal cell suspension and serum levels of ALT (G) of mice challenged with i.p. injection of 300 mg/kg of acetaminophen in WT C57BL/6 or protease-activated receptor 2 (PAR2)-deficient mice. The data are combined results of 4 experiments and represented as means ±SEM (*n* =  total number of mice from the multiple experiments for each group). * *P*<0.05, NS; not significant versus WT groups. PBS groups; WT mice received i.p. PBS.

Since dead cells release proteases, we hypothesized that PAR2 might be involved in inflammatory responses to dying cells. We evaluated this hypothesis by injecting necrotic EL4 cells i.p. into PAR2-deficient or wild type mice and quantifying the neutrophil influx into the peritoneum at 4 and 18 hours. The neutrophil influx was reduced in the PAR2-mutant mice compared to wild type animals at the early time point but not at the late time point (Fig. 2A, B, C, D).

We also induced hepatocyte necrosis in PAR2-deficient and wild type mice with acetaminophen and then quantified the ensuing inflammatory response. The neutrophilic infiltration stimulated by this form of injury was reduced in the livers of the PAR2 mutant mice, whereas monocyte recruitment was not decreased (Fig. 2E, F). The magnitude of liver injury did not differ in PAR2 mutant mice from wild type (Fig. 2G). These results demonstrate that PAR2 participates in cell death-induced neutrophil recruitment at least in the early phase in two distinct assay systems.

### Role of complement in cell death-induced inflammation

Complement activation can stimulate inflammation through the generation of two proinflammatory mediators, C3a and C5a [32], [33]. These two soluble complement fragments are generated by the hydrolysis of the third and fifth components of complement. Complement proteins have been demonstrated to deposit onto dead cells in tissues, indicating that cell death can trigger the complement cascade [34]. Therefore, complement activation has the potential to contribute to cell death-induced inflammation and this mechanism has been implicated in stimulating inflammation in a model of reperfusion injury [35].

**Figure 3:**
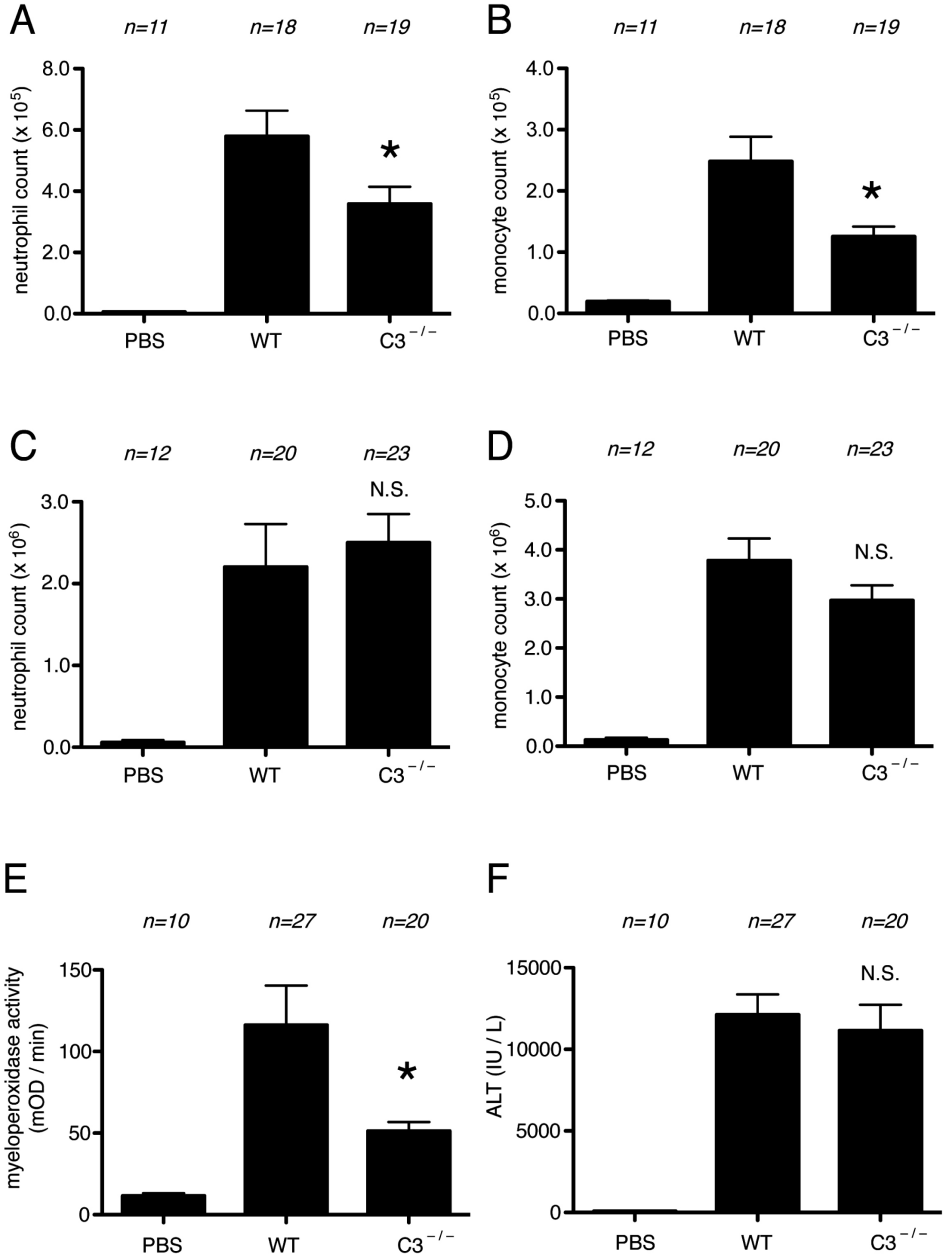
Role of C3 in cell-death induced inflammation. (A, B) The total neutrophil (A) and monocyte (B) numbers in peritoneal cavity of WT C57BL/6 mice and C3-deficient mice 4 hours after i.p. injection of heat-shocked necrotic EL4 cells. (C, D) The total neutrophil (C) and monocyte (D) numbers in peritoneal cavity of WT C57BL/6 mice and C3-deficient mice 16 hours after i.p. injection of heat-shocked necrotic EL4 cells. (E, F) Myeloperoxidase activity in the liver (E) and serum levels of ALT (F) of mice challenged with i.p. injection of 300 mg/kg of Acetaminophen in C57BL/6 or C3 deficient mice. The data are combined results of 5 experiments and represented as means ±SEM (*n* =  total number of mice from the multiple experiments for each group). * *P*<0.05, NS, not significant versus WT groups. PBS groups, WT mice received i.p. PBS.

To further investigate the contribution of complement to the cell death-induced inflammatory response, we utilized mice that genetically lacked C3 [25]. Such mice cannot generate C3a. Moreover, since C3 cleavage is required to activate the downstream components of the pathway (including C5 in most situations), these mice will generally not produce C5a [36]. We injected necrotic EL4 cells or saline into the peritoneum of wild type or C3-mutant mice and then measured the influx of neutrophils into this body cavity. The recruitment of neutrophils and monocytes to cell death was significantly reduced in mice deficient in C3, compared to wild type ones (Fig. 3A, B) at the early, 4 hour time point. In contrast, at the peak of the response, 16 hours later [8], [9] the inflammatory response to dead cells was similar in C3-deficient and wild type mice (Fig. 3C, D).

### Role of antibody in cell death-induced inflammation

It has been proposed that injured cells may activate complement when they release intracellular components such as nonmuscle myosin that are bound by natural antibodies [22]. The resulting immune complexes could trigger complement activation via the classical pathway. In addition, antibodies might help trigger inflammation in other ways. For example, natural antibodies can promote the nucleation of uric acid, a known DAMP released from dying cells, into its proinflammatory form (monosodium urate crystals) [37]. Therefore, we investigated whether mice that genetically lacked antibodies (µMT) had any reduction in inflammation to injected dead cells or drug-induced liver injury.

**Figure 4:**
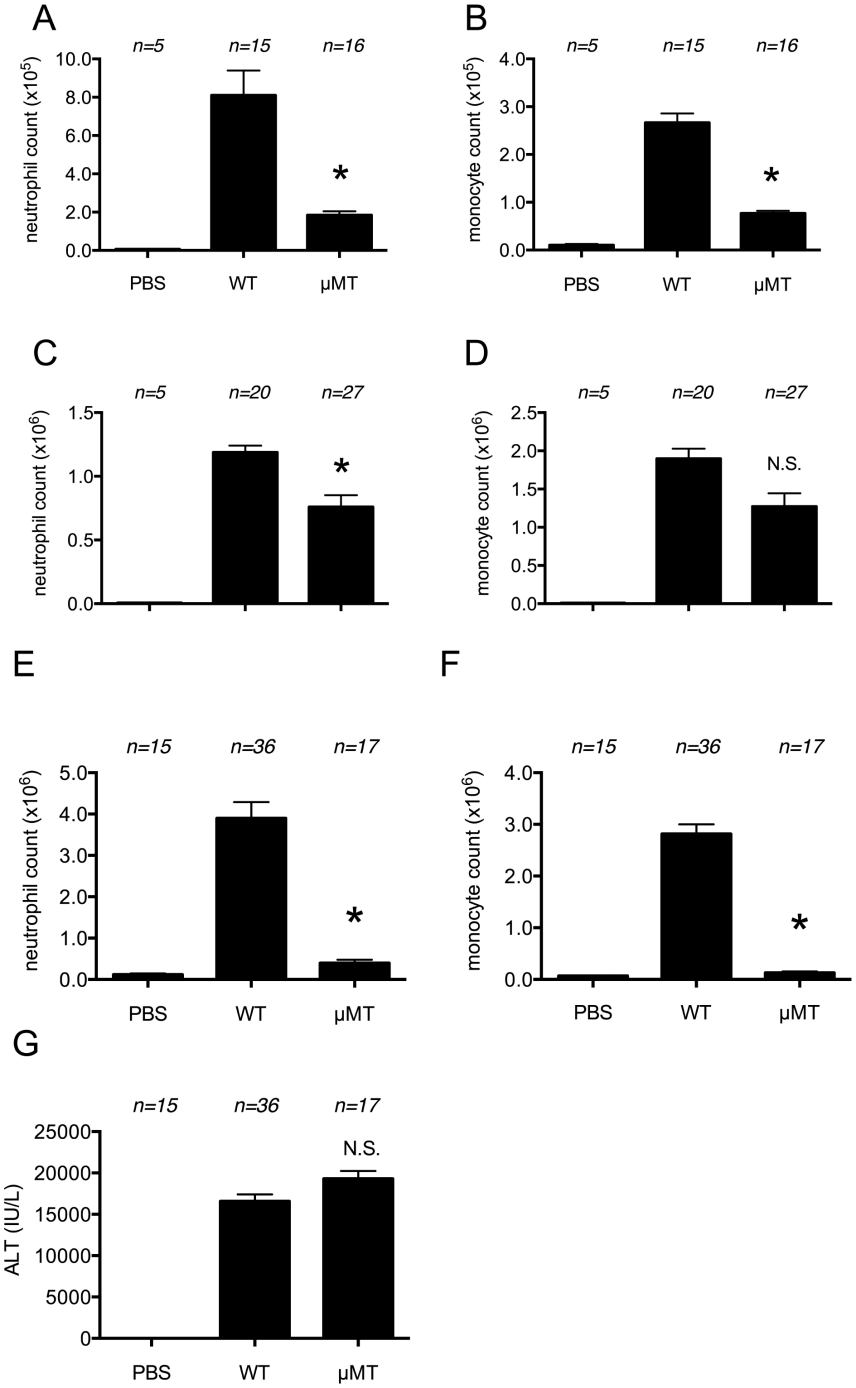
Role of antibody in cell-death induced inflammation. (A, B) The total neutrophil (A) and monocyte (B) numbers in peritoneal cavity of WT C57BL/6 mice and B lymphocyte-deficient µMT mice 4 hours after i.p. injection of heat-shocked necrotic EL4 cells. (C, D) The total neutrophil (C) and monocyte (D) numbers in peritoneal cavity of WT C57BL/6 mice and µMT mice 16 hours after i.p. injection of heat-shocked necrotic EL4 cells. (E, F, G) The total neutrophil (E) and monocyte (F) numbers in hepatic nonparenchymal cell suspension and serum levels of ALT (G) of mice challenged with i.p. injection of 300 mg/kg of acetaminophen in WT C57BL/6 or B lymphocyte-deficient µMT mice. The data are combined results of 5 experiments and represented as means ±SEM (*n* =  total number of mice from the multiple experiments for each group). * *P*<0.05, NS; not significant versus WT groups. PBS groups; WT mice received i.p. PBS.

Four hours after receiving an injection of dead EL4 cells i.p., µMT mice showed a significant reduction in both of neutrophil and monocyte inflammation compared to wild type animals (Fig. 4A, B). The reduction in inflammation persisted at 16 hours (Fig. 4 C, D). Similarly, there was a reduction in the neutrophilic infiltration in the liver of acetaminophen-treated µMT mice compared to wild type ones whereas the intensity of liver injury in µMT mice was similar to that wild type ones (Fig. 4E, F, G).

**Figure 5:**
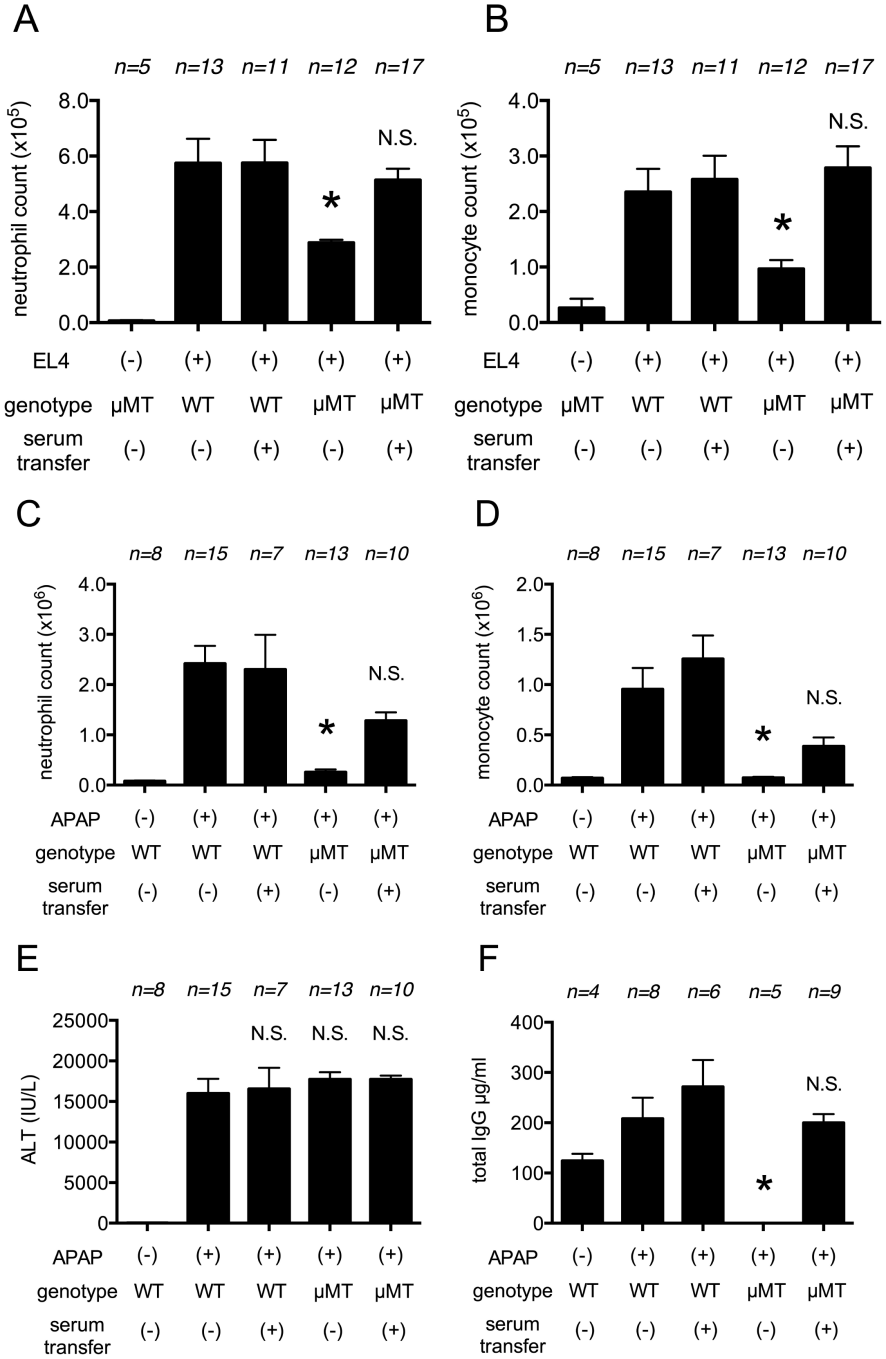
Restoration of cell-death induced inflammation by antibody-containing normal serum transfer. (A, B) The total neutrophil (A) and monocyte (B) numbers in peritoneal cavity of WT C57BL/6 mice and B lymphocyte-deficient µMT mice with or without preceding reconstitution of antibody-containing normal serum 4 hours after i.p. injection of heat-shocked necrotic EL4 cells. (C, D, E, F) The total neutrophil (C) and monocyte (D) numbers in hepatic nonparenchymal cell suspension and serum levels of ALT (E) and of total IgG (F) of mice challenged with i.p. injection of 300 mg/kg of acetaminophen in WT C57BL/6 or B lymphocyte-deficient µMT mice. The data are combined results of 4 experiments and represented as means ±SEM (*n* =  total number of mice from the multiple experiments for each group). * *P*<0.05, NS; not significant versus WT groups. PBS groups; WT mice received i.p. PBS.

To further investigate whether the absence of antibody was responsible for the reduced cell death-induced inflammation in µMT mice, we transferred antibody-containing normal mouse serum into the µMT mice. The inflammation induced by necrotic EL4 cells injected i.p. was significantly increased by serum transfer (Fig. 5A, B). Similarly, serum transfer also significantly increased acetaminophen-induced hepatic inflammation in the µMT mice (Fig. 5C, D, E, F).

### Role of ATP in cell death-induced inflammation

ATP released from dying cells has been implicated as a “find me” signal, at least for cells undergoing apoptosis [38]. It has also been suggested that ATP from necrotic cells functions as a proinflammatory DAMP [19]. Consistent with this idea, ATP stimulates previously activated macrophages through their P2X7 receptors to produce IL-1, at least *in vitro* [24], [39], and IL-1 is a key cytokine in eliciting the cell death-induced inflammatory responses [9].

**Figure 6:**
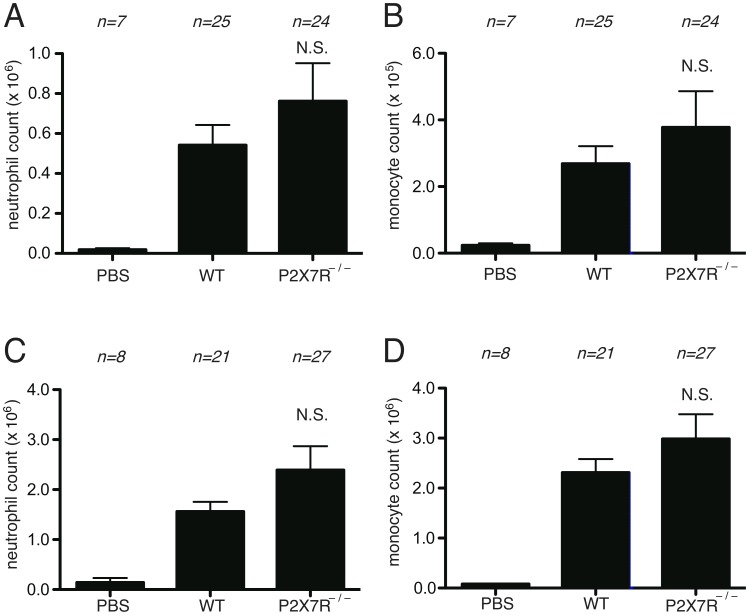
Role of ATP in cell-death induced inflammation in peritoneal cavity. (A, B) The total neutrophil (A) and monocyte (B) numbers in the peritoneal cavity of WT C57BL/6 mice and P2X7 receptor –deficient mice 4 hours after i.p. injection of heat-shocked necrotic EL4 cells. (C, D) The total neutrophil (C) and monocyte (D) numbers in peritoneal cavity of WT C57BL/6 mice and P2X7 receptor –deficient mice 16 hours after i.p. injection of heat-shocked necrotic EL4 cells. The data are combined results of 5 experiments and represented as means ±SEM (*n* =  total number of mice from the multiple experiments for each group). NS, not significant versus WT groups. PBS groups, WT mice received i.p. PBS.

To investigate the contribution of the ATP- P2X7 pathway to cell-death induced inflammation, we injected dead cells into the peritoneum of wild type or P2X7-deficient mice and quantified the ensuing inflammation at the peak of responses. The neutrophil influx into the peritoneum was similar in both the wild type and mutant animals (Fig. 6A, B). Inflammation was also not reduced in P2X7-deficient mice at early time points (Fig. 6C, D).

**Figure 7:**
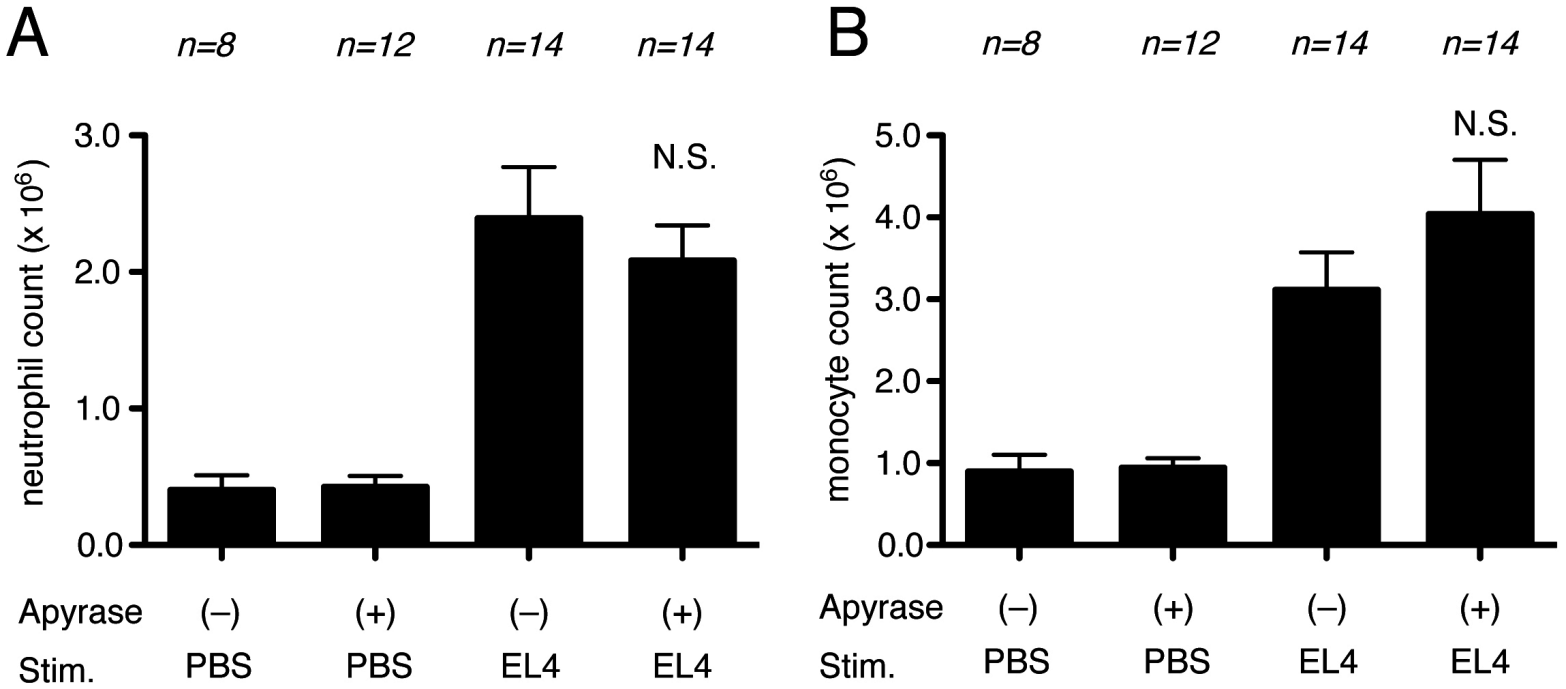
Role of ATP in cell-death induced inflammation. The total neutrophil (A) and monocyte (B) numbers in the peritoneal cavity of WT C57BL/6 mice 16 hours after i.p. injection of heat-shocked necrotic EL4 cells with or without apyrase. The data are combined results of 3 experiments and represented as means ±SEM (*n* =  total number of mice from the multiple experiments for each group). NS, not significant versus WT groups stimulated with necrotic EL4 only.

It is possible to deplete extracellular ATP *in vivo* by hydrolysis with the enzyme apyrase. Therefore, in another set of experiments we investigated the effect on inflammatory responses of injecting i.p. apyrase together with dead cells. Consistent with the results of the experiments with P2X7-deficient mice, apyrase did not inhibit the inflammatory response to dead cells (Fig 7A, B). Injection of apyrase by itself did not induce inflammation and therefore was not masking any effect of the hydrolysis of ATP.

**Figure 8:**
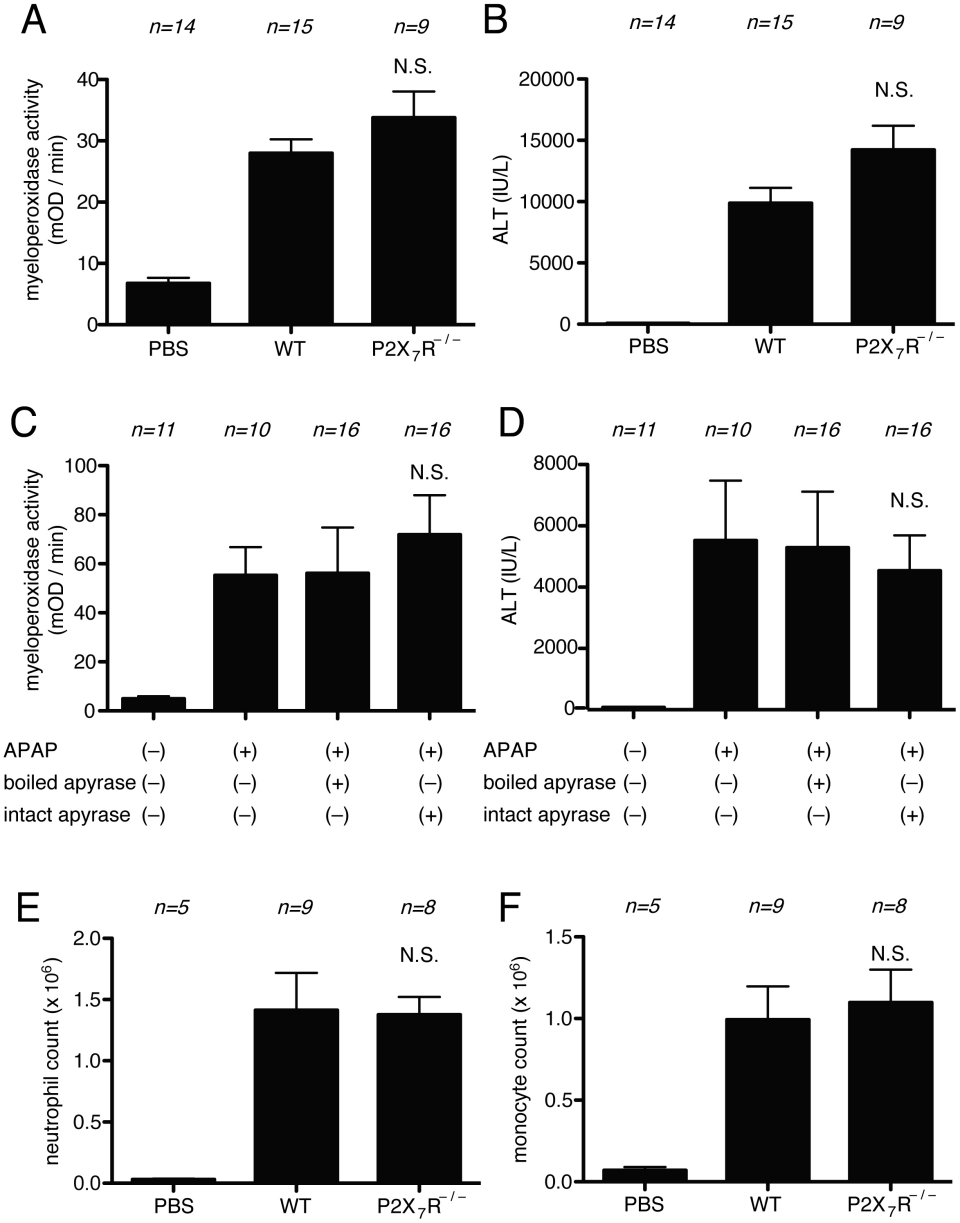
Role of ATP in cell-death induced inflammation in Acetaminophen-liver toxicity. (A, B) Myeloperoxidase activity in the liver (A) and serum levels of ALT (B) of mice challenged with i.p. injection of 300 mg/kg of Acetaminophen in C57BL/6 or P2X7R deficient mice. (C, D) Myeloperoxidase activity in the liver (C) and serum levels of ALT (D) of mice challenged with i.p. injection of 300 mg/kg of Acetaminophen in intact or boiled apyrase treated C57BL/6 mice. (E, F) The total neutrophil (E) and monocyte (F) numbers in the peritoneal cavity of WT C57BL/6 mice and P2X7 receptor -deficient mice 16 hours after i.p. injection of liver homogenate. The data are combined results of 2–3 experiments and represented as means ±SEM (*n* =  total number of mice from the multiple experiments for each group). NS, not significant versus WT groups (A, B, E, F). NS, not significant versus boiled apyrase groups (C, D). PBS groups, WT mice received i.p. PBS.

To confirm and extend these results, we also examined the contribution of the ATP- P2X7 pathway on the inflammatory response to drug-induced liver injury. In these experiments there was no significant reduction in inflammation to acetaminophen injection in P2X7-deficient mice or in mice injected with apyrase (Fig 8A, B, C, D). Therefore, we find no evidence for a major role of ATP in cell death-induced inflammation in two distinct experimental models.

These results were surprising because it was previously reported that hydrolyzing ATP or eliminating its receptor (P2X7) reduced inflammation to a laser-induced thermal injury in the liver [19]. We hypothesized that the difference in results between these models could be that the cells killed immediately by thermal injury might release greater amounts of ATP, whereas the drug-injured liver and heat-shocked EL4 may have depleted their intracellular stores of this nucleotide before disruption of their plasma membrane. To test this hypothesis we rapidly disrupted normal healthy liver cells and injected these dead cells i.p. into mice. However, we again found that the inflammatory response was not reduced in P2X7-deficient mice (Fig. 8E, F). Similar results were also found when we injected non-stressed EL4 cells that were rapidly killed by mechanical disruption.

**Figure 9:**
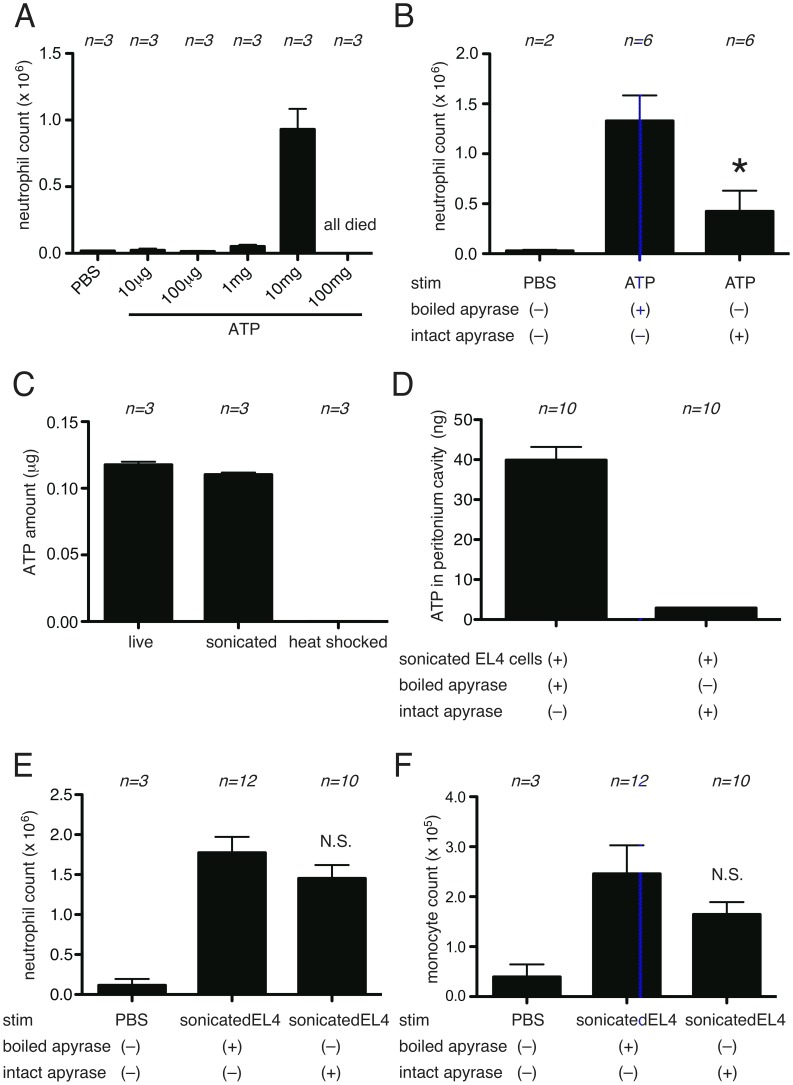
The amount of ATP in EL4 cells is less than that induces inflammation in the peritoneal cavity. (A) The total neutrophil numbers in the peritoneal cavity of WT C57BL/6 mice 4 hours after i.p. injection of indicated amount of ATP. All mice died by 4 hours in the 100 mg of ATP injected mice. (B) The total neutrophil numbers in the peritoneal cavity of WT C57BL/6 mice 4 hours after i.p. injection of boiled or intact ATP followed by injection of 10 mg of ATP. (C) The amount of ATP in 10 million of live, freshly disrupted by ultrasound sonication or heat shocked EL4 cells. (D,E,F) WT C57BL/6 mice were i.p. injected with freshly disrupted EL4 cells with boiled or intact apyrase. 4 hours later, the lavage was collected from the peritoneal cavity and the total amount of ATP (D), total numbers of neutrophil (E) and monocyte (F) are measured. The data are represented as means ±SEM (*n* =  total number of mice from the multiple experiments for each group). * *P*<0.05, NS; not significant versus boiled apyrase groups.

These results led us to directly access the proinflammatory potential of purified ATP *in vivo*. For this purpose ATP was injected i.p. into mice and the recruitment of leukocytes into the peritoneum was measured. Injection of ATP induced inflammation, but this response required relatively large amounts of the nucleoside (10 mg/mouse)(Fig. 9A). Pretreatment of mice with apyrase inhibited the inflammatory response to ATP (Fig. 9B). This control demonstrated that ATP is responsible for stimulating the inflammation and also served as a positive control that the apyrase treatment was effective *in vivo*. Given this result we analyzed the amount of ATP present in the inoculum of dead cells we were injecting and in the peritoneum post injection (Fig. 9C). EL4 cells killed by heat shock had very low levels of ATP, presumably because they hydrolyzed their intracellular pools of the nucleoside. In unstressed EL4 cells subjected to mechanical necrosis, there was 12 µg of ATP in the cells at the time of injection and after injection 40 ng of ATP in total was measured in the peritoneum (Fig. 9D). In addition, pretreatment of mice with apyrase did not significantly inhibit the inflammatory response to mechanically disrupted EL4 cells (Fig. 9E, F), further confirming that the ATP amount in the mechanically disrupted cells is less than that induces inflammation.

These amounts of ATP are below the threshold for triggering inflammation, which helps explain why ATP is a DAMP that is not required in our systems.

## Discussion

In the unperturbed state, living cells do not stimulate inflammation. However, if cells die by necrosis, then they are recognized by the innate immune system and this recognition triggers an inflammatory response [3]–[5]. This is seen in tissues whenever there is significant necrosis. Therefore, there are changes that occur upon death that somehow expose signals that are proinflammatory. In general terms it is clear that dying cells expose or release components (damage associated molecular patterns or DAMPs) that directly stimulate innate immunity or ones that indirectly lead to the generation of proinflammatory triggers. However, what these components are, what receptors they trigger and which of these molecules/receptors are most important are key issues that are incompletely understood.

Identifying the triggers of inflammation is important because the cell death-induced inflammatory response has both positive and negative effects of medical significance [4]. On the one hand, these inflammatory responses rapidly deliver host defenses to the site of injury. Once in the tissues these innate defenses then attempt to eliminate or wall off injurious agents, remove dead cells and debris, and stimulate repair. On the other hand, the immune effector components release molecules, such as reactive oxygen species and hydrolases, that causes collateral damage in the tissue and mediators that can stimulate pathological repair processes leading to fibrosis or smooth muscle hyperplasia. Through these effects the death-induced inflammatory response underlies the pathogenesis of a number of diseases [3]–[5] Therefore it is important to understand how cell death is recognized and triggers inflammation.

We have recently shown that uric acid is a proinflammatory DAMP that is released from dying cells and contributes significantly to cell death-induced inflammation in several settings, including in the peritoneum and liver models studied in the present report [18]. However, uric acid is not the only DAMP involved in these responses because its depletion reduces but does not completely eliminate inflammation. There are many candidates for other DAMPs and signals that participate in this response, including ones that bind antibody and activate complement, Mincle, PAR2 and P2X7 receptors. In this report we evaluate the contribution of these pathways to the cell death-induced inflammatory response in the same experimental systems.

It was observed many years ago that complement components were deposited onto necrotic cells *in vivo* [40]. It is thought that this occurs because the classical complement cascade is triggered when natural IgM antibodies bind to lipids exposed on the surface of dead cells, such as such as lysophosphatidylcholine [41] or to released cellular antigens, such as nonmuscle myosin [22]. It has also been suggested that proteases released from dying cells may trigger complement [42]. Once the complement cascade is activated it generates mediators that can stimulate inflammation. The most important of these proinflammatory mediators are C3a and C5a, which are soluble fragments generated from the proteolytic cleavage of C3 and C5 [32], [33]. C3 is upstream of C5 and an essential component for the classical pathway of complement activation that is triggered by antibodies. However, the importance of complement activation to cell death-induced inflammation is incompletely understood.

Our results indeed show a role for complement in the cell death-induced inflammatory response *in vivo*. The death-induced inflammatory response is partially but significantly attenuated in mice that lack the C3 complement component compared to wild type ones (Fig. 3A, B). C3 is required for all of the major pathways of complement activation (classical, lectin and alternate pathways) and in all these pathways is needed for the activation of the distal components of the cascade, including C5. Therefore it is likely that the magnitude of the reduction in inflammation in the C3-deficient mice defines the extent of the contribution of complement to the cell death-induced inflammatory response, although it is possible that there could be some activation of C5 through some other mechanism [43].

Surprisingly, C3 was not required at later time points of the cell death-induced inflammatory response to dead EL4 cells (Fig. 3C, D). This may indicate that while complement may be one of the earliest mediators, at later time points other mediators are generated that are sufficient and dominate the response and/or that the complement activation is very transient. This contrasts with the role of complement in inflammation in an ischemia reperfusion injury [42], [44], where its contribution appears to be sustained. Perhaps these differences are due to the nature and amount of DAMPs released in the different settings and/or how long complement activation is sustained.

Since antibodies may help trigger complement activation to dying cells, it was of interest to evaluate their potential contribution to cell death-induced inflammatory responses. Since antibodies are generated by B lymphocytes, we examined the inflammatory response to cell death in mice lacking antibody due to a genetic deficiency in B lymphocytes. These experiments revealed that both neutrophilic and monocytic inflammation triggered by dead cells was reduced in mice lacking B cells and antibody (Fig. 4). Moreover, these responses could be substantially restored by antibody–containing serum transfer (Fig. 5). Therefore, antibodies appear to contribute to cell death-induced inflammation and presumably do so by activation of complement.

Our findings showed one difference between the contribution of complement and that of B cells and/or antibody in the peritoneum. Complement was only required at early time points while B cells/antibodies participated at both early and late time points. Therefore, at the later time point B cells/antibodies are contributing to cell death-induced inflammation via a complement independent mechanism(s). What this mechanism is, is not known; however one possibility is that it could be antibody augmentation of the proinflammatory signal imparted by uric acid, a DAMP released from dying cells. Uric acid has been shown to contribute to the inflammatory response to dying cells at the 18 hours time point [18] and natural anti-urate antibodies have been implicated in promoting the nucleation of uric acid into crystals and thereby promoting inflammation [37]. Alternatively, it is possible that antibodies are engaging Fc receptors on leukocytes and thereby triggering inflammation.

Another way dying cells have been reported to stimulate inflammation is by engaging the C-type lectin receptor Mincle [21]. Mincle is a leukocyte receptor that binds a number of microbial PAMPs and when triggered induces inflammation. Previous studies have found that inflammation to thymic irradiation is inhibited by administration of anti-Mincle antibody [21]. It was proposed that dying cells release the endogenous DAMP SAP130 that then binds to and stimulates Mincle. To test the role of Mincle in our system we used Mincle-deficient mice instead of antibody blockade. Surprisingly, we found no reduction in cell death-induced inflammation in animals that totally lack Mincle, in both liver and peritoneal models and at early and peak time points (Fig. 1). Why there is a discrepancy in the role of Mincle in our studies versus earlier ones is unclear. It is formally possible that the Mincle-deficient mouse has some compensatory change. Alternatively, and we suspect more likely, antibodies can have pleotropic effects that can influence results. In the case of Mincle, this receptor is expressed on the responding leukocytes (including neutrophils) and it is possible that the anti-Mincle antibody is inhibiting inflammation through antibody effector mechanisms, such as target cell depletion, rather than through an inhibition of Mincle function. Yet another possibility is that Mincle plays a role in some responses but not others.

PAR2 is another receptor whose properties made it a candidate to participate in the cell death-induced inflammatory response. This receptor is expressed on leukocytes and is activated when its ectodomain is cleaved by proteases to generate a soluble stimulatory ligand. When this ligand is liberated it binds to the PAR2 receptor causing the production of inflammatory mediators. Since endogenous cellular proteases can activate PAR2, and such proteases are released from dying cells, we hypothesized that PAR2 might participate in the inflammatory response to cell death, and found that this response was indeed reduced in PAR2-deficient mice (Fig. 2). Like Complement, we could only detect significant role for PAR2 in the early inflammatory response in the peritoneum. Therefore, PAR2 is required for cell death-induced inflammatory responses, at least in our assay systems.

Another potential proinflammatory DAMP is ATP. Cells contain intracellular pools of ATP and this molecule can be released from necrotic cells as their plasma membrane loses integrity. ATP stimulates macrophages through their P2X7 receptor and this is a potent stimulus for activation of the NLRP3 inflammasome leading to the generation and secretion of mature (bioactive) IL-1β [39], [45]. ATP also functions as a “find me” signal guiding phagocytes to apoptotic cells [38]; however apoptotic cells are often non-inflammatory and also their release of ATP requires caspase-mediated activation of the pannexin channel, which should not occur in necrosis. Despite these known effects of ATP, in our systems we find no reduction in cell death-induced inflammation in animals that genetically lack the P2X7 receptor or that have been treated with apyrase to hydrolyze free ATP (Fig. 6, 7, 8). Therefore, we conclude that ATP is not a major contributor to the cell death-induced inflammatory response, at least in our systems.

Our results with ATP were surprising because a previous study found that ATP depletion with apyrase or eliminating the P2X7 receptor reduces cell death-induced inflammation in a laser-induced liver injury model [19]. Presumably, this difference in results is somehow related to differences in the type of injury. One possibility is that this is due to the actual cell types that are injured and releasing ATP. In the laser liver injury model, all cell types in the illumination field will be killed, including hepatocytes, Kuppfer cells, stellate cells and stromal elements. In contrast in the drug-induced liver injury model the cell that is injured is only the hepatocytes. Another possibility is that cells contain and release more ATP in the thermal liver injury model than in the acetaminophen-injury model. Indeed it has been shown that ATP levels in hepatocytes can decrease after acetaminophen treatment [46]–[48]. While these mechanisms may account for the difference between the models, we still found no reduction in inflammation to the injection of liver subjected to rapid mechanical necrosis; in this situation the dying cell won't metabolize its stores of ATP and the nucleotide is rapidly released (Fig. 8E, F).

In any case, our finding that ATP is not required for cell-death induced inflammation should not be surprising. In many situations where cells are mortally injured, they rapidly degrade their intracellular stores of ATP. A good example of this is ischemic injury, where ATP levels plummet rapidly in the affected cells [49]. Despite this loss of ATP such ischemic tissue injury stimulates a robust inflammatory response *in vivo*. In fact, this response is so characteristic that the presence of inflammation is used clinically to diagnose and date the time of tissue infarction [50]. Our findings with heat-shocked EL4 cells are another such example.

One benefit of our studies is that we have compared the role of multiple potential DAMPs in the same experimental systems. We have previously found a contribution from uric acid but not from some other putative DAMPs such as HMGB1 [8] or in most situations from IL-1 released from the dying cell [9]. Based on the present study we have shown that complement, B cells/antibody and PAR2 are contributors to these responses but that ATP, Mincle are dispensable. In future studies it will be important to define what other DAMPs and receptors participate in these responses.
