# Correction: Evaluation of the Contribution of Multiple DAMPs and DAMP Receptors in Cell Death-Induced Sterile Inflammatory Responses

**DOI:** 10.1371/journal.pone.0113029

**Published:** 2014-11-04

**Authors:** The PLOS ONE Staff

Dr. Yoshitaka Kimura should be included in the author byline. Dr. Kimura should be listed as the fourth author, and is affiliated with 2: the Department of Internal Medicine, Teikyo University School of Medicine, Tokyo, Japan and with the Department of Allergy and Rheumatology, the University of Tokyo Graduate School of Medicine. The contributions of this author are as follows: conceived and designed the work, acquired, analysed, and interpreted data, drafted the article and revised it for intellectual content, approved the final version for publication, agreed to be accountable for all aspects of the work.

The correct citation is: Kataoka H, Kono H, Patel Z, Kimura Y, Rock KL (2014) Evaluation of the Contribution of Multiple DAMPs and DAMP Receptors in Cell Death-Induced Sterile Inflammatory Responses. PLoS ONE 9(8): e104741. doi:10.1371/journal.pone.0104741
